# Knowledge, acceptability, and attitudes regarding minimally invasive tissue sampling (MITS) techniques in Brazil

**DOI:** 10.1371/journal.pgph.0006147

**Published:** 2026-04-13

**Authors:** Livia Mendes Almeida, Deborah Nunes Melo, Pedro Mansueto Melo de Souza, Tania Mara Silva Coelho, Anacelia Gomes de Matos Mota, Juliana Costa de Abreu Leite, Barbara Feitosa Leite, Mariana Mendonça Quezado, Beatriz Santos de Castro Gadelha, Fernando Barroso Duarte Filho, Jesamar Correia Matos Filho, Beatriz Oliveira Caetano, Marina Marques Maia, Ana Teresa Morais Martins, Rogier van Doorn, Jennifer Ilo Van Nuil, Ngan Ta Thi Dieu, Luciano Pamplona de Góes Cavalcanti

**Affiliations:** 1 Faculdade de Medicina, Universidade Christus, Brasil; 2 Programa de Pós-graduação em Patologia, Universidade Federal do Ceará, Brasil; 3 Serviço de Verificação de Óbitos Dr Rocha Furtado, Secretaria de Saúde do Ceará, Brasil; 4 Secretaria de Saúde do Estado do Ceará, Brasil; 5 Oxford University Clinical Research Unit, Hanoi and Ho Chi Minh City, Vietnam; 6 Nuffield Department of Medicine, University of Oxford, Oxford, United Kingdom; PLOS: Public Library of Science, UNITED STATES OF AMERICA

## Abstract

Determining cause of death is fundamental for health services to guide the formulation of appropriate public policies to reduce and prevent mortality. In Brazil, some deaths still do not have their underlying cause defined, and in these cases, performing a complete diagnostic autopsy (CDA) is essential. However, there are challenges that hinder its performance, and in this scenario, minimally invasive tissue sampling (MITS) could be a promising alternative. Here, we explored the knowledge, acceptability, and attitudes towards CDA and MITS in Northeast Brazil. We conducted a cross-sectional study using structured questionnaires that were disseminated using snowball sampling through medical societies networks, social networks, instant messaging groups, and email. Chi-square tests or Fisher’s exact tests were performed, as appropriate, to determine the difference between the five groups (i.e., pathologists, non-pathologist physicians, other health professionals, medical students, and the general population). 1,519 individuals participated, predominantly female (67.9%) with a median age of 41 (range 21 – 83) years. There was widespread recognition of the importance of CDA (79.6%) and a predisposition to authorize it among family members (67.0%). 1.5% reported knowing about MITS, and 83.4% believed that greater publicity for MITS would increase accessibility. Among physicians and pathologists (n = 141), 52.2% agreed that trained professionals could perform the technique, and 79.3% agreed that MITS had a lower cost and required less hands-on time than CDA. Regarding implementation, blood (52.2%) and liver (26.1%) were identified as the easiest organs to sample, while brain (50.0%) and spleen (24.0%) were considered the most technically difficult. Although widely accepted among scholars in the field, MITS is little known outside of this environment. Investments in training, standardization of protocols for consent and conduct, and communication strategies that are sensitive to the sociocultural context are fundamental for its adoption as a complementary tool in determining the cause of death in countries like Brazil.

## Introduction

Determining cause of death is fundamental for health services to guide appropriate public health policies to prevent premature mortality and expand medical knowledge with more assertive interventions. Knowledge about cause of death enables not only therapeutic strategies, but also public health policies, better planning for the purchase of medication, and more targeted surveillance and immunization programs, especially in the case of infectious diseases [1].

Complete diagnostic autopsy (CDA) is considered the gold standard for establishing cause of death, and recent studies show that understanding the aetiology of death from pneumonia, encephalitis, and dengue-like syndromes, for example, can provide valuable insights into specific etiological agents [2,3]. However, the number of CDAs conducted has decreased in recent decades in several countries, including Brazil. Among the factors related to this decline are family refusal, religious and cultural barriers, as well as structural and human resource limitations [4,5].

In contexts such as these, the minimally invasive tissue sampling (MITS) technique could provide a promising alternative to CDA. MITS consists of using thick hollow needles to collect fragments of organs and body fluids by puncture, with or without the use of portable ultrasound [6–9]. Studies have already demonstrated that MITS shows good diagnostic agreement with CDA, especially in cases involving infectious and emerging diseases [5,10–12].

In the last decade, there has been an increase in publications on MITS, reflecting technological and methodological advances that have consolidated its diagnostic potential [4,7,9,10,13]. Despite this, its incorporation into pathology services and routine surveillance still faces resistance, which varies according to professional experience, infrastructure availability, and local sociocultural factors [14]. In studies comparing MITS to CDA, participants indicated that both healthcare professionals and the families of the deceased highlighted several favourable aspects of MITS including an interest in knowing the cause of death, the speed of the procedure, and the smaller marks left by needle puncture incisions [15–17].

Others emphasized that acceptance of the technique increases when it is preceded by clear and respectful communication between professionals and family members [18]. Healthcare professionals reported that adequate training, well-defined protocols, and institutional trust are crucial for the sustainable implementation of MITS [19].

In Brazil, recent advances include the creation of a protocol for the use of MITS in the Death Verification Service (SVO) in Ceará, in Northeast Brazil, accompanied by investments in laboratory infrastructure and training of pathologist physicians [20]. Despite this progress, little is known about the acceptance of the technique outside of the clinical setting. International literature has already reinforced the importance of understanding the perceptions of health professionals to enable its incorporation into health systems [14], but in the Brazilian context, gaps remain regarding the views of different audiences, including medical professionals.

Considering that the adoption of MITS depends not only on technical competence but also on social acceptance, it becomes essential to investigate how physicians, other health professionals, and the general population perceive the use of MITS when CDA is not possible. This study constitutes a first step in a broader initiative to strengthen the use of the MITS technique by evaluating the knowledge, acceptability, and attitudes among physicians, health professionals, and the population residing in Northeast Brazil, and by identifying barriers and facilitators for its future implementation.

## Methods

We conducted an analytical cross-sectional study in Northeast Brazil

### Ethical Statements

The study was conducted in accordance with Resolution No. 466/2012 of the National Health Council, Brazil. This study was approved by the Ethics Committee by Unichristus through registration CAAE 27162619.10000.5049, with Opinion number: 3.851.684. All participants gave their consent electronically before the start of the questionnaire.

### Population and sample

Individuals residing in the State of Ceará, aged ≥18 years and who agreed to participate in the research by signing the Informed Consent Form (ICF), were included.

Five distinct groups of participants were recruited: 1-pathologist physicians, 2 - physicians with other areas of specialization, 3 - other health professionals, 4 - medical students, and 5 - general population. Among medical students, we subdivided participants between those who had completed up to the eighth semester and those who were in their internship. Questionnaires with duplicate responses or those with less than 80% completion were excluded.

### Data collection procedures

We developed a structured questionnaire elaborated from a literature review [1] and adapted based on the sociocultural context of Brazil for this study. The questionnaires were administered in two phases. First, the pathologist physicians were invited to participate after being sensitized through the Brazilian Society of Pathology/Ceará, an entity that includes all 50 registered pathologist physicians active in the State. Data were collected between January and March 2025 through an electronic form disseminated by the Society and reinforced through the use of the snowball sampling techniques [21,22].

The second phase involved the other four groups, and data collection took place in May 2025. The questionnaires were disseminated through social networks, instant messaging groups, institutional email lists, and snowball sampling to reach different audiences [21,22].

At the beginning of each questionnaire, there were questions common to all groups, and when participants identified themselves in each of the designated groups, a second stage with relevant questions for that group began.

### Research instrument (S1 Questionnaire)

The questionnaire included Yes/No, single-choice, and multiple-choice questions, as well as those presented on a five-point Likert scale (from “strongly disagree” to “strongly agree”). The questions addressed knowledge, acceptability, and attitudes regarding CDA and MITS, as well as possible barriers to implementation of MITS.

The questionnaire contained blocks of questions that addressed: 1) sociodemographic data (age, sex, education, profession/specialty, and religion); 2) experience with death (occurrence of death in the family and perception of knowledge of the cause); 3) prior knowledge about CDA and MITS; 4) attitudes and acceptability of the procedure in different population groups (neonates, children, pregnant women, adults, and the elderly); 5) perceived barriers (body marks caused by procedures, religion, delay in releasing the body, lack of information about the procedure, trust in clinical diagnoses); and 6) perceived benefits (discovery of hereditary diseases, elucidation of the cause of death, contribution to science and teaching).

### Data analysis

Data analysis was conducted using IBM SPSS Statistics 25 software. Quantitative variables were reported as mean and standard deviation, while qualitative variables were illustrated as frequencies and percentages. The chi-square test or Fisher’s exact tests were performed appropriately to determine the difference between groups.

## Results

### Sample characterization

The study included 1,519 individuals, comprising 499 (32.8%) medical students, 385 (25.3%) general population, 364 (24.0%) healthcare professionals, 249 (16.4%) non-pathologist physicians, and 22 (1.4%) pathologist physicians. The median age was 34 (18–83) years, and was lower among medical students (21.5%) and higher among the general population and other medical specialties (41.0%). With the exception of pathologist physicians, in all other groups, female sex (67.9%) and a completed higher education level (61.5%) predominated. Catholicism predominated in all groups, ranging from 50.9% for the general population to 75.1% among physicians. When asked whether they had heard about a CDA, most respondents (95.5%) said yes. For all physician group, the percentage was 100% and 91.2% for the general population. Regarding MITS, knowledge was only 100% among pathologists and between 13.4% and 32.6% in all other groups (Table 1).

**Table 1:** Sociodemographic characteristics and knowledge about CDA and MITS among participants. Ceará, Brazil, 2025.

Variable	Total (n=1.519)	Pathologists (n=22)	Other physicians (n=249)	Healthcare professionals (n=364)	Medical students (n=499)	General public (n=385)
**Age** **Median [range]**	-	40 [32–72]	37 [22–83]	41 [19–79]	21.5 [16–55]	41 [14–77]
**Sex N (%)**						
Female	1032 (67.9)	7 (31.8)	159 (63.9)	293 (80.7)	320 (64.1)	253 (65.7)
Male	486 (31.9)	15 (68.2)	90 (36.1)	70 (19.3)	179 (35.9)	132 (34.3)
**Religion N (%)**						
Catholic	971 (63.9)	16 (72.7)	187 (75.1)	220 (60.4)	352 (70.5)	196 (50.9)
Protestant Christian	210 (13.8)	1 (4.5)	15 (6.0)	57 (15.7)	72 (14.4)	65 (16.9)
Spiritist	84 (5.5)	1 (4.5)	7 (2.8)	29 (8.0)	15 (3.0)	32 (8.3)
Other / No religion	222 (14.6)	4 (18.3)	40 (16.1)	44 (12.1)	46 (9.2)	88 (22.9)
**Education N (%)**						
High school	60 (4.0)	-	-	-	-	60 (15.6)
College (not completed)	499 (32.8)	-	-	-	499 (100.0)	-
College (completed)	333 (21.9)	1 (4.5)	16 (6.6)	-	-	316 (82.1)
Specialist	329 (21.7)	8 (36.4)	118 (48.6)	203 (55.8)	-	-
M Sc / Ph D	272 (17.9)	12 (54.5)	99 (40.7)	161 (44.3)	-	-
**Medical students N (%)**						
First 2 years	-	-	-	-	254 (50.8)	-
Last two years	-	-	-	-	102 (38.4)	-
Intern	-	-	-	-	53 (10.8)	-
**Heard of complete diagnostic autopsy N (%)**	1450 (95.5)	22 (100.0)	249 (100.0)	362 (99.5)	466 (93.4)	351 (91.2)
**Heard of MITS N (%)**	327 (21.5)	22 (100.0)	70 (28.1)	118 (32.6)	70 (15.0)	47 (13.4)

**Legend:** Values are expressed as absolute numbers and percentages. Age is presented as median and minimum and maximum values. MITS = Minimally Invasive Tissue Sampling; CDA = Complete Diagnostic Autopsy.

### Knowledge and perceptions about CDA

More than 90% of respondents considered the performance of CDA important, and most of these participants considered the greatest benefit to be the clarification of the cause of death (83.6%), especially in cases of explaining sudden death (71.0%). They also reported that it was easier to authorize CDA in adults (63.8%), followed by the elderly (58.5%) and neonates (41.5%). Possible emotional discomfort was the most cited reason for not allowing CDA for 59.3% of respondents. The mother (35.7%) and spouses (29.7%) were reported to be most responsible for this authorization (Table 2).

**Table 2:** Knowledge, acceptability, and perceptions about CDA and MITS among participants. Ceará, Brazil, 2025.

Question	Answer	Total (%)	Physicians (%)	Other Healthcare professionals (%)	Medical students (%)	General population (%)
**How do you value the importance of autopsies in healthcare?**	Very important	79.6	80.7	76.9	80.9	80.1
	Important	17.5	16.5	17.6	17.8	18.5
	unnoted	2.6	2.8	5.5	1.3	1.1
**If a close relative died without a clear cause, would you allow an autopsy?**	Yes	67.0	69.5	65.9	66.1	66.8
	Don’t know	19.8	18.5	20.6	21.0	18.2
	No	13.2	12.0	13.5	12.8	15.1
**In what situations can an autopsy be performed?**	Sudden death	71.0	78.3	70.1	72.6	69.8
	Unknown cause	67.1	65.4	68.4	63.2	67.0
	Suspected medical error	47.7	48.2	52.5	45.3	46.1
**Who in the family would likely make the decision to authorize an autopsy?**	Spouse	29.7	39.0	36.8	23.0	20.1
	Mother	35.7	31.6	30.1	38.6	42.5
	Father	26.1	22.9	27.5	25.8	28.4
**In which age group would an autopsy be most acceptable?**	Adults	63.8	65.5	67.3	60.1	63.0
	Elderly	58.5	58.6	61.5	55.9	59.2
	Neonates	41.5	41.2	38.9	45.5	40.3
**What reasons can lead to the refusal of an autopsy by patient/ family?**	Emotional distress	59.3	58.9	62.5	61.4	54.2
	Religious reasons	49.5	51.6	47.5	50.7	57.4
	Lack of information	43.1	40.5	41.2	38.6	42.3
**Do you think it’s necessary to know the cause of death?**	Yes	91.9	91.9	90.7	93.4	91.2
**What benefits would the family have in allowing an autopsy?**	Knowing the cause of death	83.6	82.4	85.7	84.2	81.6
	Emotional tranquillity	70.2	69.5	73.6	70.5	67.4
	Scientific contribution	47.3	55.1	48.9	44.7	40.8
**Have you heard of MITS?**	Yes	20.4	28.1	32.6	15	13.4
**Do you believe more autopsies would be performed if people had more information about MITS?**	Yes	83.4	87.1	83.0	84.2	80.3
**Groups where MITS is more acceptable (3 most cited)**	Adults	60.0	62.2	65.0	52.4	57.0
	Children	52.5	53.4	51.6	55.8	49.3
	Neonates	54.4	58.1	54.2	60.0	44.8
**Would individuals who would refuse a full autopsy more easily accept MITS?**	Yes	75.5	77.5	74.4	78.8	70.9

**Legend:** Values are presented as absolute numbers (percentages), calculated based on the total number of valid responses in each group for each question. In multiple-choice items (groups where MITS is more acceptable), only the three most frequently cited reasons are shown.

Slightly over 20% of respondents had heard of MITS, but after clarification, more than 75% of those who would not authorize CDA would allow MITS for a relative; mainly in deaths involving adults. The majority of participants (83.4%) believed that more MITS procedures would be performed if people had more information about MITS (Table 2).

**Fig 1:**
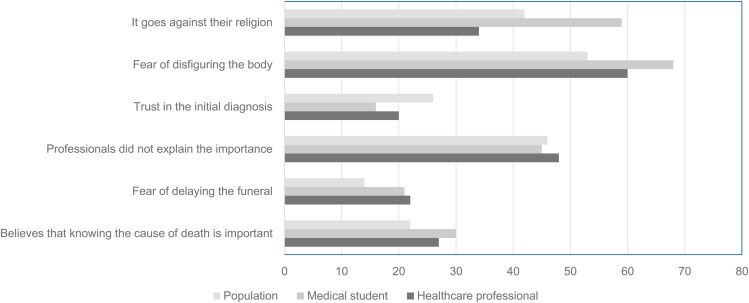
Reasons given for possible refusal of autopsy by relatives.

The main reasons given for not authorizing a CDA were fear of disfiguring the deceased’s body, lack of explanation of the procedure’s importance, and religious reasons (Fig 1). Among physicians, the profile was different. The greatest limitations reported were not related to cultural or religious beliefs, but rather to technical and operational issues. More than half (59.1%) of the participants stated that they did not feel fully qualified to perform MITS outside of a Death Verification Service, highlighting the lack of practical training as the main obstacle. Furthermore, 41% of the physicians cited difficulties in communicating with family members as a barrier to accepting the technique, and many reinforced the importance of clear terminology, preferring the term “MITS” to “minimally invasive autopsy - MIA,” a term used in Ceará when the technique was first introduced.

### Physicians’ Knowledge and Perceptions Regarding CDA and MITS

Slightly over a third of pathologists (36.4%) believed that other medical specialties could be trained and should perform MITS. Similar values were found when questioned about other health professions (Table 3). Most physicians agreed that MITS should have a lower cost (85.7%) than CDA and would require less time to perform (95.6%). Pathologists, however, believed there would be no difference between the cost of the two techniques, and slightly more than half believed it would be a faster procedure. When asked which type of organ should be the easiest to reach using MITS, they reported blood, liver, and lungs. They considered the brain and spleen more difficult (Table 3).

**Table 3:** Physicians’ perception and knowledge, by specialty, regarding MITS. Fortaleza, Brazil, 2025.

Questions	Categories	Total	Pathologists	Other Physicians
**Could any physician perform MITS?**	Yes	52.2	36.4	57.1
	No	39.1	63.6	31.4
	I don’t know	8.7	–	11.4
**Is MITS more costly than CDA?**	Yes	20.7	40.9	14.3
	No	79.3	59.1	85.7
**Does performing MITS take longer than CDA?**	Yes	12.0	36.4	4.3
	No	88.0	63.6	95.7
**Organs considered easiest to reach with MITS**	Blood	52.2	40.9	55.7
	Liver	26.1	31.8	24.3
	Lung	10.9	13.6	10.0
**Organs considered most difficult to reach with MITS**	Brain	50.0	36.4	54.3
	CSF	11.0	–	12.9
	Heart	–	–	10.0
	Spleen	24.0	45.5	–
**Should other health professionals, provided they are trained, perform MITS?**	Yes	57.6	36.4	64.3
	No	42.4	63.6	35,7

**Legend:** Data are expressed in absolute numbers and percentages, considering the total number of valid responses in each professional category. CSF = cerebrospinal fluid.

Considering only pathologists who had or had not undergone training on MITS, there was no significant difference between the organs considered easiest to access (p = 0.37) and the organs considered most difficult to access (p = 0.78) using MITS. Most participants (72.7%) believed that family acceptance also depended on the terminology used for the procedure, preferring “Minimally Invasive Tissue Collection - MITS” to “Minimally Invasive Autopsy - MIA”. Although familiarity with MITS was universal, only 10 pathologists (45.5%) had received formal training, which was more common among those who currently worked or have worked in a Verification of Death Service (p = 0.03).

## Discussion

The results of this study show that, although CDA is widely recognized as an essential tool for clarifying the cause of death and for scientific advancement, its performance still faces cultural, emotional, and operational barriers. More than 80% of participants attributed high importance to CDA, which reinforces its historical role in the construction of medical knowledge [5,15].

However, emotional discomfort, the influence of religious beliefs, and lack of information were identified as the main factors of resistance, especially among medical students and the general population—a finding consistent with studies conducted in different sociocultural contexts [17,18]. On the other hand, our study revealed a curious finding. Most of the respondents reported their religion as Catholic or Protestant Christian, and these are religions that, in principle, do not cite any opposition to these autopsy procedures. These results indicate that current challenges are not only technical but also related to communication, highlighting the importance of educational and professional training strategies that promote more empathetic and transparent approaches with family members.

MITS could be a viable alternative to CDA, especially in contexts where logistical, religious, or cultural factors limit the execution of CDA. However, knowledge about the technique proved to be limited. Approximately only 20% of those who completed the questionnaire had heard of MITS, although more than 80% believed that after more communication and dissemination would increase the number of MITS performed. This pattern was similar to that observed by Das et al. [14] and Ben-Sasi et al. [23], who highlight the lack of knowledge as one of the main obstacles to the implementation of MITS in hospital and community settings. Among medical students, familiarity with the method was almost non-existent, limited to the students who had completed more advanced semesters. This result suggests the need to include these topics earlier in the medical training curriculum.

MITS is widely perceived as a less invasive, lower-cost procedure with reduced execution time—a perception shared by more than 79% of the physicians and pathologists in this study. These results are consistent with the findings of Duarte-Neto et al. [8] and Wagensveld et al. [11], who validated the accuracy and efficiency of the technique in determining the cause of death in adults. Although 52.2% of participants believe that non-pathologist physicians could perform MITS with adequate training, the pathologists themselves emphasized the need for specific training, highlighting one of the main limitations for its large-scale implementation. This perception converges with the observations of Paganelli et al [9], who emphasize the importance of standardizing protocols and rigorous technical training to ensure diagnostic quality. This divergence of opinions reflects both legitimate concerns about methodological consistency and an opportunity to strengthen inter-professional dialogue. Successful experiences in countries such as Uganda and Mozambique demonstrate that the integration between pathologists and clinical teams, supported by continuous training and structured protocols, is fundamental for the effective implementation of MITS [5].

Regarding acceptability, both CDA and MITS were more accepted to be conducted in adults and the elderly, and less so in neonates. This pattern reflects the affective and symbolic dimension attributed to the infant body, in line with findings by Maixenchs et al. [24] and Das et al. [14], who identified greater refusal in pediatric cases due to the affective burden and the perception of prolonged suffering. In contrast, MITS showed potential to mitigate some of this discomfort, as it is perceived as less invasive and preserves bodily appearance and better aesthetic preservation — factors considered decisive in family acceptance [17,19].

The benefits attributed to MITS were widely recognized by all groups. Highlighted benefits included the discovery of hereditary diseases (70–77%), the elucidation of the cause of death (65–70%), and the contribution to science and teaching (55–59%). In addition, about 60% of participants pointed to the technique as a tool for preventing future deaths, by improving the understanding of transmissible diseases or hereditary conditions. Among pathologists, the perceived benefits were even more objective. In addition to those already mentioned, they reported the speed of the procedure, lower operating costs, and the preservation of bodily integrity, emphasizing that these factors would increase practical viability and the chance of obtaining family consent. They recognized MITS as a valuable alternative to CDA in contexts that require minimal invasiveness and rapid release of the body but emphasized its limitations — particularly in the detection of focal or macroscopically apparent lesions, such as acute myocardial infarction or tumors, even with the use of ultrasound.

The perception of the cost and duration of the procedure was largely favorable to MITS, considered faster and less expensive by almost 80% of the participants. This aspect is relevant in low- and middle-income countries, where the scarcity of human resources and infrastructure limits the performance of complete autopsies [9]. Studies conducted in different resource-limited contexts reinforce that MITS, in addition to being logistically more feasible, can generate diagnostic results comparable to those of complete autopsy, especially when applied in a standardized way and in the case of infectious diseases [8,11].

The organs most frequently cited in this study as being the easiest to access — blood, liver, and lungs — coincide with the main tissues used in international MITS protocols for etiological determination of the cause of death [5,15]. This convergence reinforces the practical perception of the method’s applicability and suggests that the professionals’ technical knowledge is aligned with international scientific recommendations. On the other hand, the identification of organs considered difficult to access, such as the brain and spleen, reveals limitations inherent to the technique, also reported by Ben-Sasi et al. (2013), who highlight the need for ultrasound support and specific training to increase sample accuracy.

The pathologists also highlighted less body disfigurement as a determining factor for family acceptance, corroborating evidence from Ben-Sasi et al. [19] and Suwalowska et al. [17]. Despite this, they reported feeling insecure about performing the procedure in a hospital setting, citing a lack of practical training and institutional protocols as the main barriers. This finding reinforces the need for investments in technical training, practical simulations, and the integration of MITS into medical residency programs, as already suggested by Paganelli et al. [9] and Rakislova et al. [5].

Finally, MITS was widely recognized as a technique with the potential to complement, not replace, conventional autopsy. Its adoption can strengthen epidemiological surveillance, generate more accurate data on causes of death, and contribute to evidence-based public policies, provided it is accompanied by social awareness and adequate institutional support [8,9,11]. The integration of MITS into the Brazilian healthcare system must, therefore, consider not only its technical feasibility, but also the cultural and communicational determinants that permeate the relationship between healthcare professionals and families, ensuring that the method is incorporated in an ethical, sustainable, and socially acceptable manner. The Death Verification Service began offering continuing education to professionals who work with autopsies, and MITS became part of the training for resident physicians.

This study has important strengths, including a large and heterogeneous sample encompassing physicians, pathologists, other health professionals, medical students, and members of the general population, which allowed for a comprehensive assessment of knowledge, acceptability, and attitudes toward CDA and MITS across different societal perspectives. In addition, the inclusion of a real-world sociocultural context from Northeast Brazil provides valuable evidence from a middle-income setting where cause-of-death ascertainment remains a public health challenge. Nevertheless, some limitations must be acknowledged. The cross-sectional design precludes causal inference, and the use of non-probabilistic snowball sampling may limit the generalizability of the findings, particularly given the overrepresentation of individuals with higher educational levels and those connected to health-related networks. Self-reported responses are also subject to recall and social desirability biases. Despite these limitations, the findings offer clear recommendations for future implementation of MITS, including: expanding structured training programs for MITS, particularly within medical education and pathology residency curricula; developing standardized institutional protocols for consent and procedure execution; and implementing culturally sensitive communication strategies aimed at families and communities. Future studies should explore longitudinal and qualitative approaches to better understand decision-making processes and assess the impact of targeted educational interventions on the sustainable integration of MITS into health surveillance systems.

## Conclusion

The results of this study demonstrate that, despite the widespread appreciation of autopsy as a diagnostic and scientific tool, cultural, emotional, and structural barriers persist that limit its implementation in the Brazilian context. MITS emerges as a promising alternative, especially as it is seen as less invasive, faster, and less costly, which may favor its acceptance among healthcare professionals and family members of the recently deceased. However, knowledge about the technique is still limited, even among physicians and medical students, reflecting the need for dissemination and training strategies. The positive perception of pathologists regarding the feasibility of MITS reinforces its potential as a complementary method to CDA, particularly in situations where performing the latter proves unfeasible. Thus, expanding knowledge about MITS among professionals and the general population, promoting specific training, and adopting more empathetic communication approaches are fundamental steps to consolidate this technique as a viable tool for improving health surveillance and the quality of information on causes of death in Brazil.

## Supporting information

S1 Questionnaire Questionnaire used in the research. (DOCX)

## References

[1] Dieu NTT, Phuong ND, Le Thao MN, Chambers M, Nguyen DM, Nguyen HTL, et al. Knowledge and attitudes toward complete diagnostic autopsy and minimally invasive autopsy: A cross-sectional survey in Hanoi, Vietnam. PLOS Glob Public Health. 2023;3(3):e0001685. doi: 10.1371/journal.pgph.0001685 36963097 PMC10022770

[2] Phuong ND, Dieu NTT, Nguyen MLT, Phuoc AL, Suwalowska H, Nguyen DM, et al. Hypothetical acceptability of minimally invasive tissue sampling and considerations for practice: A qualitative study in Vietnam. Glob Public Health. 2024;19(1):2403097. doi: 10.1080/17441692.2024.2403097 39284583

[3] de Souza WM, Fumagalli MJ, de Lima STS, Parise PL, Carvalho DCM, Hernandez C. Pathophysiology of chikungunya virus infection associated with fatal outcomes. Cell Host Microbe. 2024;32(4):606-622.e8. doi: 10.1016/j.chom.2024.02.011 PMC11018361 38479396

[4] Maixenchs M, Anselmo R, Martínez Pérez G, Oruko K, Agnandji ST, Angoissa Minsoko PC, et al. Socio-anthropological methods to study the feasibility and acceptability of the minimally invasive autopsy from the perspective of local communities: lessons learnt from a large multi-centre study. Glob Health Action. 2019;12(1):1559496. doi: 10.1080/16549716.2018.1559496 30712476 PMC6366403

[5] Rakislova N, Fernandes F, Lovane L, Jamisse L, Castillo P, Sanz A, et al. Standardization of minimally invasive tissue sampling (MITS) and histology procedures across seven CHAMPS sites in Africa and Asia. Clin Infect Dis. 2019;69(Suppl 4):S302–S310. doi: 10.1093/cid/ciz565 PMC6785668 31598667

[6] Byass P. Minimally Invasive Autopsy: A New Paradigm for Understanding Global Health?. PLoS Med. 2016;13(11):e1002173. doi: 10.1371/journal.pmed.1002173 27875535 PMC5119692

[7] Bassat Q, Castillo P, Martínez MJ, Jordao D, Lovane L, Hurtado JC, et al. Validity of a minimally invasive autopsy tool for cause of death determination in pediatric deaths in Mozambique: An observational study. PLoS Med. 2017;14(6):e1002317. doi: 10.1371/journal.pmed.1002317 28632739 PMC5478091

[8] Duarte-Neto AN, de Almeida Monteiro RA, Johnsson J, dos Passos Cunha M, Pour SZ, Saraiva AC. Ultrasound-guided minimally invasive autopsy during the 2018 São Paulo yellow fever epidemic: correlation with conventional autopsy. PLoS Negl Trop Dis. 2019;13(7):e0007625. doi: 10.1371/journal.pntd.0007625 PMC6675127 31329590

[9] Paganelli CR, Goco NJ, McClure EM, Banke KK, Blau DM, Breiman RF, et al. The evolution of minimally invasive tissue sampling in postmortem examination: a narrative review. Glob Health Action. 2020;13(1):1792682. doi: 10.1080/16549716.2020.1792682 32713325 PMC7480574

[10] Palhares AEM, Ferreira L, Freire M, Castillo P, Martínez MJ, Hurtado JC, et al. Performance of the minimally invasive autopsy tool for cause of death determination in adult deaths from the Brazilian Amazon: an observational study. Virchows Arch. 2019;475(5):649–58. doi: 10.1007/s00428-019-02602-z 31201504 PMC6861203

[11] Wagensveld IM, Weustink AC, Kors JA, Blokker BM, Hunink MGM, Oosterhuis JW. Effect of minimally invasive autopsy and ethnic background on acceptance of clinical postmortem investigation in adults. PLoS One. 2020;15(5):e0232944. doi: 10.1371/journal.pone.0232944 32392247 PMC7213690

[12] Melo DN, Lima GRP, Fernandes CG, Teixeira AC, Filho JB, Araújo FMC, et al. Post-Mortem Diagnosis of Pediatric Dengue Using Minimally Invasive Autopsy during the COVID-19 Pandemic in Brazil. Trop Med Infect Dis. 2022;7(7):123. doi: 10.3390/tropicalmed7070123 35878135 PMC9316822

[13] Castillo P, Martínez MJ, Ussene E, Jordao D, Lovane L, Ismail MR, et al. Validity of a Minimally Invasive Autopsy for Cause of Death Determination in Adults in Mozambique: An Observational Study. PLoS Med. 2016;13(11):e1002171. doi: 10.1371/journal.pmed.1002171 27875530 PMC5119723

[14] Das MK, Arora NK, Kaur G, Malik P, Kumari M, Joshi S, et al. Perceptions of family, community and religious leaders and acceptability for minimal invasive tissue sampling to identify the cause of death in under-five deaths and stillbirths in North India: a qualitative study. Reprod Health. 2021;18(1):168. doi: 10.1186/s12978-021-01218-4 34348749 PMC8336381

[15] Maixenchs M, Anselmo R, Zielinski-Gutiérrez E, Odhiambo FO, Akello C, Ondire M, et al. Willingness to Know the Cause of Death and Hypothetical Acceptability of the Minimally Invasive Autopsy in Six Diverse African and Asian Settings: A Mixed Methods Socio-Behavioural Study. PLoS Med. 2016;13(11):e1002172. doi: 10.1371/journal.pmed.1002172 27875532 PMC5119724

[16] Rugwizangoga B, Niyibizi JB, Ndayisaba MC, Musoni E, Manirakiza F, Uwineza A. Exploring perceptions and acceptance of minimally invasive tissue sampling among bereaved relatives and healthcare professionals in Rwanda: a qualitative study. BMJ Open. 2021;11(12):e049767. 34938081 10.2147/JMDH.S340428 PMC8685444

[17] Suwalowska H, Kingori P, Parker M. Navigating uncertainties of death: perceptions of minimally invasive autopsy. Glob Public Health. 2023;18(1):2180065. doi: 10.1080/17441692.2023.2180065 36853068 PMC9988304

[18] Otieno P, Akelo V, Khagayi S, Omore R, Akoth K, Nyanjom M. Acceptability of minimally invasive autopsy by community members and healthcare workers in western Kenya. PLOS Glob Public Health. 2023;3(9):e0001319. doi: 10.1371/journal.pgph.0001319 PMC10519588 37747874

[19] Ben-Sasi K, Chitty LS, Franck LS, Thayyil S, Judge-Kronis L, Taylor AM, et al. Ben-Sasi K, Chitty LS, Franck LS, Thayyil S, Judge-Kronis L, Taylor AM, et al. Acceptability of a minimally invasive perinatal/paediatric autopsy: healthcare professionals’ views and implications for practice. Prenat Diagn. 2013;33(4):307–12. doi: 10.1002/pd.4077 23457031

[20] Almeida LM de, Melo DN de, Silva MM da, Souza PMM de, Silva FK de S, Coelho TMS, et al. Usefulness of minimally invasive autopsy in the diagnosis of arboviruses to increase the sensitivity of the Epidemiological Surveillance System in Ceará, Brazil. Epidemiol Serv Saude. 2024;33:e2024008. doi: 10.1590/S2237-96222024V33E2024008.en 38808901 PMC11131572

[21] Sudman S, Freeman HE. The use of network sampling for locating the seriously ill. Med Care. 1988;26(10):992–9. doi: 10.1097/00005650-198810000-00007 3172869

[22] Siddiqui NA, Rabidas VN, Sinha SK, Verma RB, Pandey K, Singh VP, et al. Snowball Vs. House-to-House Technique for Measuring Annual Incidence of Kala-azar in the Higher Endemic Blocks of Bihar, India: A Comparison. PLoS Negl Trop Dis. 2016;10(9):e0004970. doi: 10.1371/journal.pntd.0004970 27681709 PMC5040448

[23] Maixenchs M, Anselmo R, Sanz A, Castillo P, Macete E, Carrilho C, et al. Healthcare providers’ views and perceptions on post-mortem procedures for cause of death determination in Southern Mozambique. PLoS One. 2018;13(7):e0200058. doi: 10.1371/journal.pone.0200058 29979720 PMC6034841

