# Supplementary material for: Knowledge, acceptability, and attitudes regarding minimally invasive tissue sampling (MITS) techniques in Brazil
Source: PLOS Glob Public Health. 2026 Apr 13;6(4):e0006147. doi: 10.1371/journal.pgph.0006147 (PMC13075663; doi:10.1371/journal.pgph.0006147)
Supplement: S1 Questionnaire — (DOCX) [file pgph.0006147.s001.docx]

***What is your field of study? (*** *Medicine, other health profession, medical student, I am not in the health field* ***)***

***Medicine***

***Healthcare professional***

*Nursing*

*Biology*

*Biomedicine*

*Physical education*

*Nursing*

*Pharmacy*

*Physiotherapy*

*Speech therapy*

*Veterinary Medicine*

*Nutrition*

*Dentistry*

*Psychology*

*Social Work*

*Occupational Therapy*

***Medical student***

***I am not a healthcare professional.***

| **Medicine** | **Healthcare professional** | **Medical student** | General population |
| --- | --- | --- | --- |
| ***Age*** *(in years):* | ***Age*** *(in years):* | ***Age*** *(in years):* | ***Age*** *(in years):* |
| ***Sex*** *(male x female)* | ***Sex*** *(male x female)* | ***Sex*** *(male x female)* | ***Sex*** *(male x female)* |
| ***Level of education***  *Specialist*  *Master's degree*  *PhD* | ***Level of education***  *Specialist*  *Master's degree*  *PhD* | ***Which semester are you currently in?***  *1, 2, 3, 4, 5, 6, 7, 8*  *boarding school* | ***Level of education***  *Fundamental*  *Average*  *Superior* |
| ***Which religion do you identify with?***  *Catholic*  *Protestant Christian*  *Spiritist*  *Buddhist*  *Jehovah's Witness*  *I have no religion.*  *Others:_______________* | ***Which religion do you identify with?***  *Catholic*  *Protestant Christian*  *Spiritist*  *Buddhist*  *Jehovah's Witness*  *I have no religion.*  *Others:_______________* | ***Which religion do you identify with?***  *Catholic*  *Protestant Christian*  *Spiritist*  *Buddhist*  *Jehovah's Witness*  *I have no religion.*  *Others:_______________* | ***Which religion do you identify with?***  *Catholic*  *Protestant Christian*  *Spiritist*  *Buddhist*  *Jehovah's Witness*  *I have no religion.*  *Others:_______________* |
|  | ***Has a family member of yours passed away without the cause of death being known?***  *Yes*  *No*  *I don't know* | ***Has a family member of yours passed away without the cause of death being known?***  *Yes*  *No*  *I don't know* | ***Has a family member of yours passed away without the cause of death being known?***  *Yes*  *No*  *I don't know* |
|  | ***Have you ever heard of an autopsy/necropsy?***  *Yes*  *No* | ***Have you ever heard of an autopsy/necropsy?***  *Yes*  *No* | ***Have you ever heard of an autopsy/necropsy?***  *Yes*  *No* |
|  | *If the answer is NO, the questionnaire stops here.* | *If the answer is NO, the questionnaire stops here.* | *If the answer is NO, the questionnaire stops here.* |
| ***How would you assess the importance of an autopsy/necropsy in clarifying the underlying cause of death?***  *Very important*  *Important*  *Average*  *Unimportant*  *Very unimportant* | ***If you've ever heard of an autopsy, how would you assess the importance of this procedure in the healthcare field?***  *Very important*  *Important*  *Average*  *Unimportant*  *Very unimportant* | ***If you've ever heard of an autopsy, how would you assess the importance of this procedure in the healthcare field?***  *Very important*  *Important*  *Average*  *Unimportant*  *Very unimportant* | ***If you've ever heard of an autopsy, how would you assess the importance of this procedure in the healthcare field?***  *Very important*  *Important*  *Average*  *Unimportant*  *Very unimportant* |
| ***If a close relative dies without the cause of death being clarified, would you agree to allow an autopsy?***  *I completely agree.*  *Agree*  *I disagree.*  *I completely disagree.*  *Undecided* | ***In your opinion, under what circumstances can an autopsy be performed?***  *(You can select more than one option)*  *With the patient's consent before their death.*  *With the consent of the patient's family/guardians, if the patient does not express a wish to do so.*  *Under the instructions of health authorities in specific situations.*  *With the concerns of family members about the patient's condition before their death.*  *By court order*  *Other (specify) : ……………* | ***In your opinion, under what circumstances can an autopsy be performed?***  *(You can select more than one option)*  *With the patient's consent before their death.*  *With the consent of the patient's family/guardians, if the patient does not express a wish to do so.*  *Under the instructions of health authorities in specific situations.*  *With the concerns of family members about the patient's condition before their death.*  *By court order*  *Other (specify) : ……………* | ***In your opinion, under what circumstances can an autopsy be performed?***  *(You can select more than one option)*  *With the patient's consent before their death.*  *With the consent of the patient's family/guardians, if the patient does not express a wish to do so.*  *Under the instructions of health authorities in specific situations.*  *With the concerns of family members about the patient's condition before their death.*  *By court order*  *Other (specify) : ……………* |
| ***In your opinion, if a relative dies and the family doesn't know the cause of death, who would likely make the final decision to authorize an autopsy?***  *Wife/Husband*  *Children of the deceased*  *Parents of the deceased*  *Brother/Sister of the deceased*  *Other (specify) : ……………* | ***If a close relative dies without the cause of death being clarified, would you agree to allow an autopsy?***  *I completely agree.*  *Agree*  *I disagree.*  *I completely disagree.*  *Undecided* | ***If a close relative dies without the cause of death being clarified, would you agree to allow an autopsy?***  *I completely agree.*  *Agree*  *I disagree.*  *I completely disagree.*  *Undecided* | ***If a close relative dies without the cause of death being clarified, would you agree to allow an autopsy?***  *I completely agree.*  *Agree*  *I disagree.*  *I completely disagree.*  *Undecided* |
| ***In your opinion, in which of these groups is the autopsy easier to accept?***  *(You can select more than one item)*  *Newborns*  *Children*  *Adults*  *Pregnant women*  *Elderly*  *Other (specify) : ……………* | ***In your opinion, if a relative dies and the family doesn't know the cause of death, who would likely make the final decision to authorize an autopsy?***  *Wife/Husband*  *Children of the deceased*  *Parents of the deceased*  *Brother/Sister of the deceased*  *Other (specify) : ……………* | ***In your opinion, if a relative dies and the family doesn't know the cause of death, who would likely make the final decision to authorize an autopsy?***  *Wife/Husband*  *Children of the deceased*  *Parents of the deceased*  *Brother/Sister of the deceased*  *Other (specify) : ……………* | ***In your opinion, if a relative dies and the family doesn't know the cause of death, who would likely make the final decision to authorize an autopsy?***  *Wife/Husband*  *Children of the deceased*  *Parents of the deceased*  *Brother/Sister of the deceased*  *Other (specify) : ……………* |
| ***In your opinion, what reasons do you believe people might have for refusing an autopsy on a relative*** *?*  *(You can select more than one item)*  *Because autopsies go against religion.*  *Because the family is afraid of mutilating the deceased.*  *Because the family is afraid of delaying the funeral.*  *Because the family is confident in the initial clinical diagnosis.*  *Because the family believes that knowing the cause of death is not important.*  *Because healthcare professionals failed to explain the importance of autopsies.*  *Other (specify) : ……………* | ***In your opinion, in which of these groups is the autopsy easier to accept?***  *(You can select more than one item)*  *Newborns*  *Children*  *Adults*  *Pregnant women*  *Elderly*  *Other (specify) : ……………* | ***In your opinion, in which of these groups is the autopsy easier to accept?***  *(You can select more than one item)*  *Newborns*  *Children*  *Adults*  *Pregnant women*  *Elderly*  *Other (specify) : ……………* | ***In your opinion, in which of these groups is the autopsy easier to accept?***  *(You can select more than one item)*  *Newborns*  *Children*  *Adults*  *Pregnant women*  *Elderly*  *Other (specify) : ……………* |
|  | ***In your opinion, is it necessary to know the reason why someone died?***  *Extremely necessary*  *Necessary*  *Average*  *Unnecessary*  *Completely unnecessary.* | ***In your opinion, is it necessary to know the reason why someone died?***  *Extremely necessary*  *Necessary*  *Average*  *Unnecessary*  *Completely unnecessary.* | ***In your opinion, is it necessary to know the reason why someone died?***  *Extremely necessary*  *Necessary*  *Average*  *Unnecessary*  *Completely unnecessary.* |
|  | ***In your opinion, what reasons do you believe people might have for refusing an autopsy on a relative*** *?*  *(You can select more than one item)*  *Because autopsies go against religion.*  *Because the family is afraid of mutilating the deceased.*  *Because the family is afraid of delaying the funeral.*  *Because the family is confident in the initial clinical diagnosis.*  *Because the family believes that knowing the cause of death is not important.*  *Because healthcare professionals failed to explain the importance of autopsies.*  *Other (specify) : ……………* | ***In your opinion, what reasons do you believe people might have for refusing an autopsy on a relative*** *?*  *(You can select more than one item)*  *Because autopsies go against religion.*  *Because the family is afraid of mutilating the deceased.*  *Because the family is afraid of delaying the funeral.*  *Because the family is confident in the initial clinical diagnosis.*  *Because the family believes that knowing the cause of death is not important.*  *Because healthcare professionals failed to explain the importance of autopsies.*  *Other (specify) : ……………* | ***In your opinion, what reasons do you believe people might have for refusing an autopsy on a relative*** *?*  *(You can select more than one item)*  *Because autopsies go against religion.*  *Because the family is afraid of mutilating the deceased.*  *Because the family is afraid of delaying the funeral.*  *Because the family is confident in the initial clinical diagnosis.*  *Because the family believes that knowing the cause of death is not important.*  *Because healthcare professionals failed to explain the importance of autopsies.*  *Other (specify) : ……………* |
|  | ***In your opinion, what benefits would the deceased's family receive from allowing an autopsy*** *?*  *(You can select more than one item)*  *Discovering hereditary diseases for early treatment of other family members.*  *Discovering infectious diseases to find new cases and provide early treatment.*  *To identify occupational diseases for the purpose of claiming insurance and compensation from the company.*  *Identifying the exact cause of death.*  *Contributing to science and saving other lives.*  *Other (specify) : ……………* | ***In your opinion, what benefits would the deceased's family receive from allowing an autopsy*** *?*  *(You can select more than one item)*  *Discovering hereditary diseases for early treatment of other family members.*  *Discovering infectious diseases to find new cases and provide early treatment.*  *To identify occupational diseases for the purpose of claiming insurance and compensation from the company.*  *Identifying the exact cause of death.*  *Contributing to science and saving other lives.*  *Other (specify) : ……………* | ***In your opinion, what benefits would the deceased's family receive from allowing an autopsy*** *?*  *(You can select more than one item)*  *Discovering hereditary diseases for early treatment of other family members.*  *Discovering infectious diseases to find new cases and provide early treatment.*  *To identify occupational diseases for the purpose of claiming insurance and compensation from the company.*  *Identifying the exact cause of death.*  *Contributing to science and saving other lives.*  *Other (specify) : ……………* |
| ***Have you ever heard of Minimally Invasive Tissue Sampling (MITS) or Minimally Invasive Autopsy (AMI)?***  *Yes*  *No* | ***Have you ever heard of Minimally Invasive Tissue Sampling (MITS) or Minimally Invasive Autopsy (AMI)?***  *Yes*  *No* | ***Have you ever heard of Minimally Invasive Tissue Sampling (MITS) or Minimally Invasive Autopsy (AMI)?***  *Yes*  *No* | ***Have you ever heard of Minimally Invasive Tissue Sampling (MITS) or Minimally Invasive Autopsy (AMI)?***  *Yes*  *No* |
| *If the answer is NO, the questionnaire stops here.* | *If the answer is NO, the questionnaire stops here.* | *If the answer is NO, the questionnaire stops here.* | *If the answer is NO, the questionnaire stops here.* |
| ***If so, through which source would you like to learn more about this method ?***  ***(You can select more than one item)***  *Doctors*  *Other health professionals*  *Autopsy experts*  *Researchers*  *Books/Journals specializing in autopsy*  *Local authority*  *Social media (WhatsApp, Instagram, TikTok)*  *TV/Radio*  *Other (specify) : ……………* | ***Do you believe that people will change their attitude towards Minimally Invasive Tissue Sampling (MITS) and more autopsies ? Would the procedures be carried out if they had more information about this procedure?***  *I completely agree.*  *Agree*  *I disagree.*  *I completely disagree.*  *Undecided* | ***Do you believe that people will change their attitude towards Minimally Invasive Tissue Sampling (MITS) and more autopsies ? Would the procedures be carried out if they had more information about this procedure?***  *I completely agree.*  *Agree*  *I disagree.*  *I completely disagree.*  *Undecided* | ***Do you believe that people will change their attitude towards Minimally Invasive Tissue Sampling (MITS) and more autopsies ? Would the procedures be carried out if they had more information about this procedure?***  *I completely agree.*  *Agree*  *I disagree.*  *I completely disagree.*  *Undecided* |
| ***Do you believe that people will change their attitude towards Minimally Invasive Tissue Sampling (MITS) and more autopsies ? Would the procedures be carried out if they had more information about this procedure?***  *I completely agree.*  *Agree*  *I disagree.*  *I completely disagree.*  *Undecided* | ***In your opinion, in which groups is Minimally Invasive Tissue Sampling (MITS) most acceptable ?***  *(You can select more than one item)*  *Newborns*  *Children*  *Adults*  *Pregnant women*  *Elderly*  *Other (specify) : ……………* | ***In your opinion, in which groups is Minimally Invasive Tissue Sampling (MITS) most acceptable ?***  *(You can select more than one item)*  *Newborns*  *Children*  *Adults*  *Pregnant women*  *Elderly*  *Other (specify) : ……………* | ***In your opinion, in which groups is Minimally Invasive Tissue Sampling (MITS) most acceptable ?***  *(You can select more than one item)*  *Newborns*  *Children*  *Adults*  *Pregnant women*  *Elderly*  *Other (specify) : ……………* |
| ***In your opinion, in which groups is Minimally Invasive Tissue Sampling (MITS) most acceptable ?***  *(You can select more than one item)*  *Newborns*  *Children*  *Adults*  *Pregnant women*  *Elderly*  *Other (specify) : ……………* | ***would individuals who do not allow a full autopsy more readily accept Minimally Invasive Tissue Sampling (MITS)?***  *Yes*  *No*  *I don't know* | ***would individuals who do not allow a full autopsy more readily accept Minimally Invasive Tissue Sampling (MITS)?***  *Yes*  *No*  *I don't know* | ***would individuals who do not allow a full autopsy more readily accept Minimally Invasive Tissue Sampling (MITS)?***  *Yes*  *No*  *I don't know* |
| ***would individuals who do not allow a full autopsy more readily accept Minimally Invasive Tissue Sampling (MITS)?***  *Yes*  *No*  *I don't know* | ***If a relative dies without the family knowing the cause of death, would you agree to authorize Minimally Invasive Tissue Sampling (MITS) ?***  *Yes*  *No*  *I don't know* | ***If a relative dies without the family knowing the cause of death, would you agree to authorize Minimally Invasive Tissue Sampling (MITS) ?***  *Yes*  *No*  *I don't know* | ***If a relative dies without the family knowing the cause of death, would you agree to authorize Minimally Invasive Tissue Sampling (MITS) ?***  *Yes*  *No*  *I don't know* |
| ***If a relative dies without the family knowing the cause of death, would you agree to authorize Minimally Invasive Tissue Sampling (MITS) ?***  *Yes*  *No*  *I don't know* | ***Please explain why you would not agree to allow a full autopsy , but would agree to allow Minimally Invasive Tissue Sampling (MITS) in a parent ?*** | ***Please explain why you would not agree to allow a full autopsy , but would agree to allow Minimally Invasive Tissue Sampling (MITS) in a parent ?*** | ***Please explain why you would not agree to allow a full autopsy , but would agree to allow Minimally Invasive Tissue Sampling (MITS) in a parent ?*** |
| ***In your opinion, who in your family would DEFINITELY be against undergoing Minimally Invasive Tissue Sampling (MITS)?***  *(You can select more than one item)*  *Wife / Husband*  *Children of the deceased*  *Parents of the deceased*  *Brother/Sister of the deceased*  *Other (specify) : ……………* | ***In your opinion, who in your family would DEFINITELY be against undergoing Minimally Invasive Tissue Sampling (MITS)?***  *(You can select more than one item)*  *Wife / Husband*  *Children of the deceased*  *Parents of the deceased*  *Brother/Sister of the deceased*  *Other (specify) : ……………* | ***In your opinion, who in your family would DEFINITELY be against undergoing Minimally Invasive Tissue Sampling (MITS)?***  *(You can select more than one item)*  *Wife / Husband*  *Children of the deceased*  *Parents of the deceased*  *Brother/Sister of the deceased*  *Other (specify) : ……………* | ***In your opinion, who in your family would DEFINITELY be against undergoing Minimally Invasive Tissue Sampling (MITS)?***  *(You can select more than one item)*  *Wife / Husband*  *Children of the deceased*  *Parents of the deceased*  *Brother/Sister of the deceased*  *Other (specify) : ……………* |
|  |  |  |  |
| ***Would you be interested in learning more about Minimally Invasive Tissue Sampling (MITS)?***  *Yes*  *No* |  |  |  |
| ***Do you believe that having the option of a MITS (Management, Treatment, and Evaluation) has a better chance of convincing family members to undergo the procedure than a conventional autopsy/necropsy?***  *Yes*  *No*  *I don't know* |  |  |  |
| ***you think any trained physician could perform a MITS, or only pathologists?***  *Yes*  *No*  *I don't know* |  |  |  |
| ***Do you think this procedure could be done in a hospital or only at a Death Verification Service (SVO)?***  *hospital*  *SVO*  *I don't know* |  |  |  |
| ***Do you think the cost of a MITS is higher than that of a conventional autopsy?***  *Yes*  *No* |  |  |  |
| ***Do you think MITS takes longer than a conventional autopsy?***  *Yes*  *No* |  |  |  |
| ***Which organ do you think should be easiest to reach using MITS?***  *Cerebrospinal fluid*  *Blood*  *Liver*  *Brain*  *Lung*  *Heart*  *Spleen*  *Kidney* |  |  |  |
| ***Which organ do you think would be the most DIFFICULT to reach using MITS?***  *Cerebrospinal fluid*  *Blood*  *Liver*  *Brain*  *Lung*  *Heart*  *Spleen*  *Kidney* |  |  |  |
| ***Do you think it's possible for other healthcare professionals, provided they are trained, to perform MITS?***  *Yes*  *No* |  |  |  |

| **1. Identification and demographic data** | | |
| --- | --- | --- |
| **Name** : _________________________________________________________________________________ | | |
| **Sex:** ( )M ( )F **Age:** _______________ |  | |
| **Type of Relationship with the SVO:_________________________________** | | |
| **2. Have you heard about the minimally invasive autopsy (MIA) technique?** | | |
| **YES** ( ) | **NO** ( ) | |
| **3. If so, where did you hear about this technique?** | | |
| **SVO** ( )  **Scientific Literature** ( )  **Books** ( ) | **National/International Congresses** ( )  **Local events ( )**  **Other: ___________________________________________** | |
| **4. Have you received any training in the use of minimally invasive autopsy (MIA) techniques?** | | |
| **YES** ( ) | **NO** ( ) | |
| **5. Have you ever done an AMI?** | | |
| **YES** ( ) | **NO** ( ) | |
| **6. Do you feel confident approaching family members for AMI authorization?** | | |
| **YES** ( ) | | **NO** ( ) |
| **7. What type of information would make you feel more confident approaching the family?** | | |
|  | | |
| **8. Do you believe that the AMI technique may be more expensive than the conventional autopsy technique?** | | |
| **YES** ( ) | | **NO** ( ) |
| **9. Do you believe that performing an AMI (Acute Mitigation Syndrome) can be more time-consuming than a conventional autopsy technique?** | | |
| **YES** ( ) | | **NO** ( ) |
| **10. Do you feel safe having an AMI (Ambulatory Medical Examination) at the SVO (Veterinary Medical Service)?** | | |
| **YES** ( ) | | **NO** ( ) |
| **11. Do you feel comfortable performing an autopsy in a hospital, in cases where the body is not brought to the Forensic Medical Institute (SVO)?** | | |
| **YES** ( ) | | **NO** ( ) |
| **12. Which of the factors described do you consider to be directly linked to the family member's decision to agree to undergo AMI? (** rank in order of importance **...)** | | |
| **Time for release of the body ( )**  **Body disfigurement ( )** | | **Religious beliefs/cultures ( )**  **Fear of organ removal to other facilities ( )** |
| **13. In your opinion, would it be easier to obtain family consent for an AMI (Acute Mitral Valve Implantation) compared to a conventional autopsy?** | | |
| **YES** ( ) | | **NO** ( ) |
| **14. In your opinion, which of the following circumstances constitute a limiting factor for conducting the AMI at the SVO?** | | |
| **Cost ( )**  **Level of knowledge among health professionals ( )**  **Lack of training and education ( )** | | **Adequate facilities ( )**  **Availability of experts to conduct the technique ( )** |
| **15. Which of the following characteristics do you consider to be the most important for facilitating the widespread use of AMI in our referral hospitals? (** *mark the three most important in order of importance* **)** | | |
| **Greater diagnostic accuracy ( )**  **Comparatively shorter procedure ( )**  **Performing the procedure as quickly as possible to facilitate the release of the body for burial and related ceremonies ( )** | | **Preservation of the body ( )**  **Community participation (support from leaders, health professionals) ( )**  **Information and transparency (clear information about the procedure) ( )** |
| **1.6 . Among the following biological samples, which one do you THINK is the EASIEST to obtain through AMI?** | | |
| **Cerebrospinal Fluid ( )**  **Blood ( )**  **Liver ( )**  **Brain ( )** | | **Lung ( )**  **Heart ( )**  **Spleen ( )**  **Kidney ( )** |
| **17. Of the following biological samples, which do you THINK is the MOST DIFFICULT to obtain through AMI?** | | |
| **Cerebrospinal Fluid ( )**  **Blood ( )**  **Liver ( )**  **Brain ( )** | | **Lung ( )**  **Heart ( )**  **Spleen ( )**  **Kidney ( )** |
| **18. Do you think other medical specialties are possible for AMI?** | | |
| **YES** ( ) | | **NO** ( ) |
| **19. Do you think it's possible for other trained healthcare professionals to perform AMI?** | | |
| **YES** ( ) | | **NO** ( ) |
| **20. On a scale of 1 to 10, how likely are you to recommend the use of the AMI technique?** | | |
|  | | |
| **21. Is there anything about AMI that you would like to comment on? Positive, negative, suggestion?** | | |
|  | | |
